# Supplementary figures and images for: Long Interspersed Nuclear Element-1 Analytes in Extracellular Vesicles as Tools for Molecular Diagnostics of Non-Small Cell Lung Cancer
Source: Int J Mol Sci. 2024 Jan 18;25(2):1169. doi: 10.3390/ijms25021169 (PMC10816871; doi:10.3390/ijms25021169)

Figure S1.

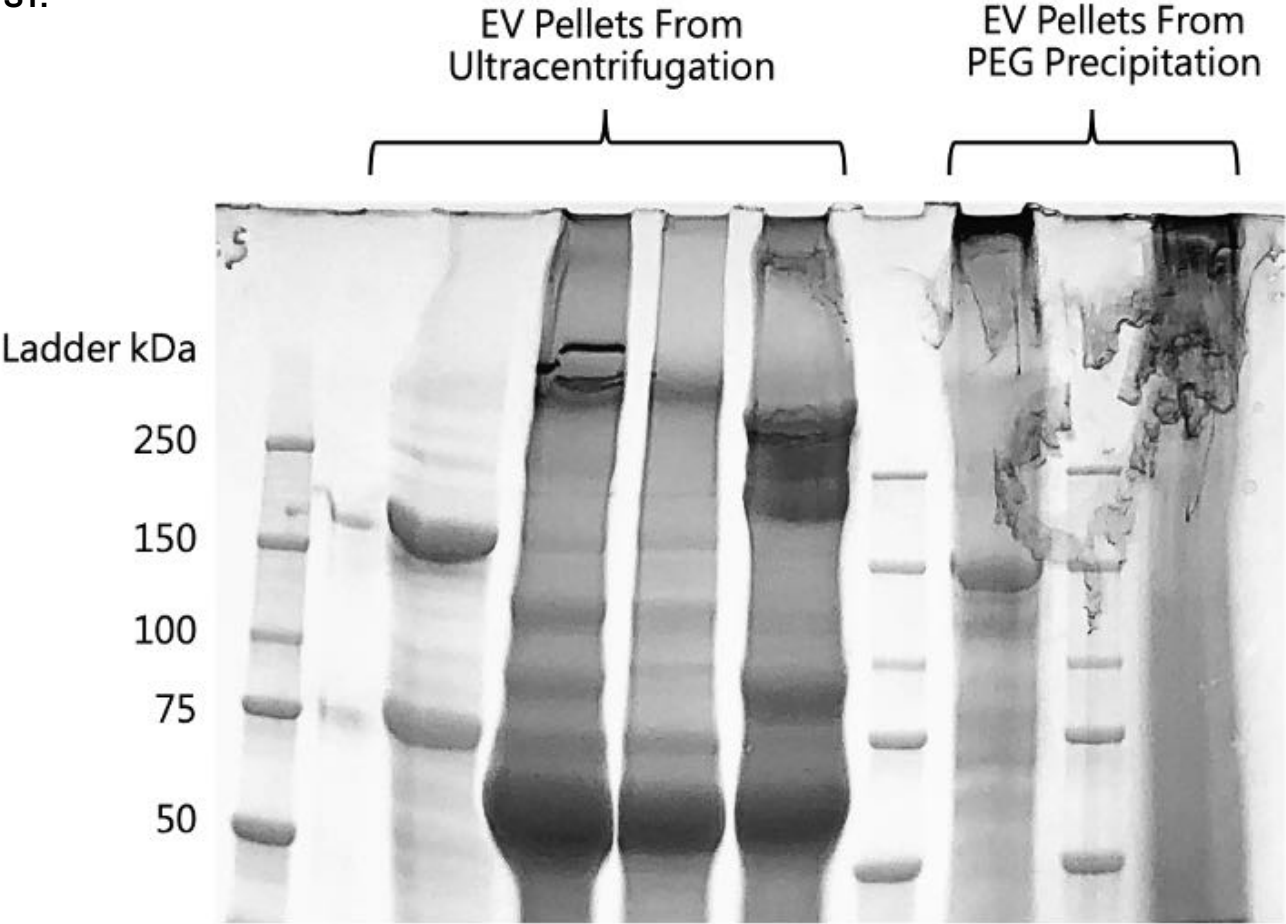

Figure S2.

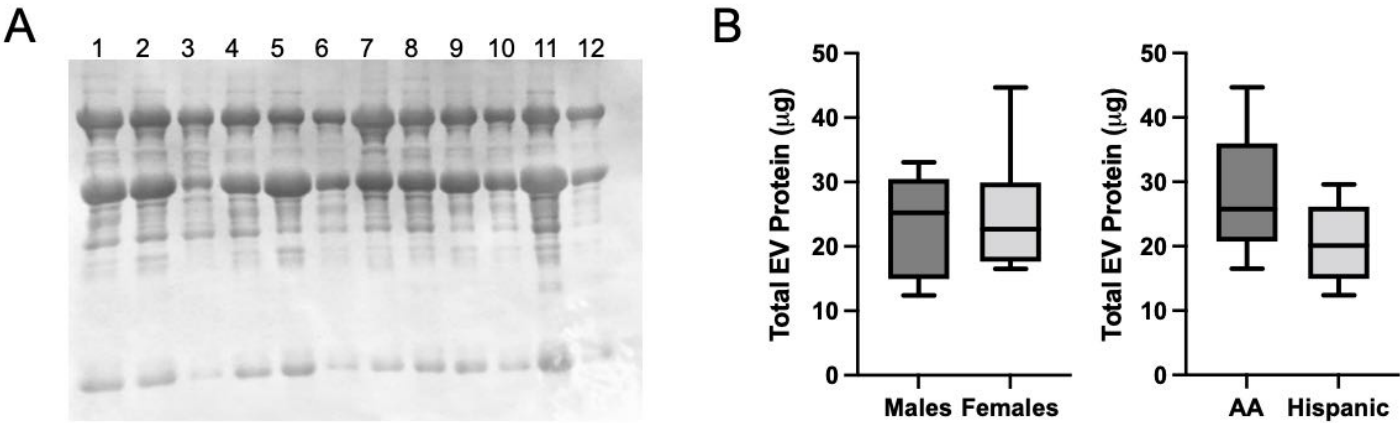

Supplement: Supplementary file 1 [file ijms-25-01169-s001.zip › ijms-2759352-supplementary.pdf]
